# Solanaceous vegetables and *Fusarium oxysporum* interactions: pathogen genomics, pathogenesis, host resistance, and emerging microbiome-driven disease management

**DOI:** 10.1093/hr/uhag074

**Published:** 2026-03-03

**Authors:** Min Li, Lihong Hao, Xinxing Shi, Jianbiao Wang, Haoqian Li, Yifei Wang, Pravin Khambalkar, Xizhe Sun, Sharmin Rima, Xinyi Guo, Xiangling Fang, Lisong Ma

**Affiliations:** State Key Laboratory of North China Crop Improvement and Regulation, Key Laboratory of Vegetable Germplasm Innovation and Utilization of Hebei, College of Horticulture, Hebei Agricultural University, Baoding 07100, China; State Key Laboratory of North China Crop Improvement and Regulation, Key Laboratory of Vegetable Germplasm Innovation and Utilization of Hebei, College of Horticulture, Hebei Agricultural University, Baoding 07100, China; State Key Laboratory of North China Crop Improvement and Regulation, Key Laboratory of Vegetable Germplasm Innovation and Utilization of Hebei, College of Horticulture, Hebei Agricultural University, Baoding 07100, China; State Key Laboratory of North China Crop Improvement and Regulation, Key Laboratory of Vegetable Germplasm Innovation and Utilization of Hebei, College of Horticulture, Hebei Agricultural University, Baoding 07100, China; State Key Laboratory of North China Crop Improvement and Regulation, Key Laboratory of Vegetable Germplasm Innovation and Utilization of Hebei, College of Horticulture, Hebei Agricultural University, Baoding 07100, China; State Key Laboratory of North China Crop Improvement and Regulation, Key Laboratory of Vegetable Germplasm Innovation and Utilization of Hebei, College of Horticulture, Hebei Agricultural University, Baoding 07100, China; Division of Plant Science, Research School of Biology, The Australian National University, ACT, Acton 2601, Australia; State Key Laboratory of North China Crop Improvement and Regulation, Key Laboratory of Vegetable Germplasm Innovation and Utilization of Hebei, College of Horticulture, Hebei Agricultural University, Baoding 07100, China; Division of Plant Science, Research School of Biology, The Australian National University, ACT, Acton 2601, Australia; State Key Laboratory of North China Crop Improvement and Regulation, Key Laboratory of Vegetable Germplasm Innovation and Utilization of Hebei, College of Horticulture, Hebei Agricultural University, Baoding 07100, China; State Key Laboratory of Grassland Agro-Ecosystems, Key Laboratory of Grassland Livestock Industry Innovation, Ministry of Agriculture and Rural Affairs, College of Pastoral Agriculture Science and Technology, Lanzhou University, Lanzhou 730020, China; State Key Laboratory of North China Crop Improvement and Regulation, Key Laboratory of Vegetable Germplasm Innovation and Utilization of Hebei, College of Horticulture, Hebei Agricultural University, Baoding 07100, China

## Abstract

Solanaceous vegetables are continuously threatened by Fusarium wilt disease, which is mainly caused by *Fusarium oxysporum* (*Fo*), a fungal species complex comprising many devastating soil-borne pathogens, resulting in severe yield losses worldwide. Over the past decade, significant and numerous advances have been made in dissecting the molecular and genomic basis underlying the interaction between solanaceous vegetables and *Fo*, particularly owing to the emergence of the tomato-*Fo* pathosystem as a powerful model system for studying the molecular basis of resistance and susceptibility in solanaceous vegetables against vascular wilt pathogens. In this review, we summarize recent advances driven by improvements in genome sequencing and assembly of *Fo* infecting solanaceous vegetables, the virulence strategies and diverse arsenals employed by *Fo* to modulate and suppress host immunity, as well as the identification and functional characterization of race-specific resistance genes in solanaceous vegetables and their corresponding *Fo* determinants. We address the potential downstream signaling pathways involved in activating solanaceous vegetable immunity against *Fo*. In addition, we explore emerging insights into microbiome-based strategies for disease control, emphasizing the potential use of beneficial and synthetic microbes in the sustainable management of Fusarium wilt in tomato. Collectively, this review provides an integrated perspective on pathogen genomics, pathogenesis, host resistance, and microbiome-driven control of Fusarium wilt in tomato, offering promising avenues for developing durable and broad-spectrum resistance against various *Fo* strains in solanaceous vegetables.

## Introduction

The *Solanaceae* family encompasses a large and evolutionarily diverse group of plants, spanning around 100 genera and approximately 2700–3000 species [1]. Members of this family include both edible and non-edible crops, together contributing substantially to the diet of humans and global agriculture economy [2]. Among edible species, it contains the most widely cultivated and economically significant horticultural crops worldwide, including potato (*Solanum tuberosum* L.), tomato (*S. lycopersicum* L.), pepper (*Capsicum* spp.), and eggplant (*S. melongena* L.). Beyond food crops, Solanaceae also comprises major non-edible crops, most prominently tobacco (*Nicotiana tabacum* L.) along with numerous species grown or harvested for medicinal, ornamental, and industrial applications [3]. However, the production of the four major solanaceous vegetable crops is increasingly threatened by Fusarium wilt, a soil-borne vascular disease caused by host-adapted formae speciales of *Fusarium oxysporum* (*Fo*), because it is nearly impossible to eradicate once established. Understanding how the pathogen adapts to different hosts, establishes infection and colonization, and how plants respond to *Fo* infection is therefore essential for elucidating disease development and for informing the design of effective management strategies.

The soil-borne vascular-wilt fungus *Fo* ranks fifth among the top 10 plant fungal pathogens [4]. It is a species complex that exhibits the ability to cause severe yield losses with a wide range of hosts, including diverse crops such as tomato, cotton, legumes, melons, and banana (plantain). Pathogenic strains of *Fo* that are capable of infecting the same host species or cultivar can be assigned to the same formae speciales (f. sp.) [5, 6]. Although formae speciales differ in host range, they often follow a comparable infection process and employ a similar infection strategy, which results in similar disease symptoms in susceptible plants [5, 7]. The infection process of Fusarium wilt begins when *Fo* penetrates the epidermis of the host root, and then *Fo* gradually grows towards the root cortex [7]. During this process, the mycelium extends towards the xylem vessels, penetrates through the pit membrane, and subsequently spreads through the roots and stems [7, 8]. Following the propagation of mycelia within xylem vessels, water transport becomes progressively restricted due to vessel blockage, resulting in yellowing of older leaves, followed by wilting, defoliation, and ultimately plant death [7]. The cross-section of infected stems typically exhibits brown discoloration of the vascular tissues [8, 9]. On the other hand, in resistant plants, fungal mycelium can penetrate the root surface and even reach the xylem, but its spread is restricted within vascular tissues, preventing systemic colonization and subsequent disease development [10].

The genome sequences of plant fungal pathogens have significantly advanced our understanding of the molecular mechanisms underlying fungal biology, pathogenesis, and host-pathogen interactions [11]. The availability of high-quality fungal genome sequences has facilitated the identification of molecular determinants that can trigger resistance gene-mediated immune responses, as well as the discovery of strategies employed by pathogens to overcome host resistance. Consequently, continuous updates on the genomic resources, including high-quality genome sequences and genome evolution of *Fo*, are indispensable for deciphering its pathogenic mechanisms, allowing us to edit host susceptibility genes to avoid *Fo* manipulation and enhance durable resistance. During infection, *Fo* secretes various molecules, including secondary metabolites, cell wall-degrading enzymes, and effectors, to facilitate colonization and disease progression [5, 12]. Among these, effectors play key roles in determining the pathogenicity of *Fo* by manipulating host cellular processes and suppressing immune responses [13]. In turn, plants have evolved plasma membrane-localized and intracellular immune receptors that sense the extracellular and intracellular immunogenic patterns associated with the pathogen infection, thereby activating downstream defense signaling [14]. Cell-surface immune receptors, such as receptor-like proteins (RLPs) and receptor-like kinases (RLKs), perceive the extracellular immunogenic patterns to activate defense responses [15]. Intracellular immune receptors are the nucleotide-binding domain and leucine-rich repeat (NLR) to detect intracellular immunogenic signals [15].

## Casual agents of Fusarium wilt diseases of solanaceous vegetables and their geographic distributions

Pepper (Capsicum spp.), potato, eggplant, and tomato are among the most widely consumed vegetables in everyday diets worldwide [16]. These vegetables are cultivated across a wide range of production systems, from field cultivation in subtropical and tropical regions to protected greenhouse [17]. However, the production of these vegetables is threatened by Fusarium wilt, which is caused by different Fusarium species. Fusarium wilt of pepper is mainly caused by host-specific *F. oxysporum* f. sp. *capsici* (*Foc*) [18]. It originated with its first report in Mexico in 1919 by Leonian [19, 20] and now this disease has been reported in many pepper-producing countries, including Russia [21], Ethiopia [22, 23], Spain [24], Egypt [25], Turkey [26], Pakistan [21], China [27], India [28], Indonesia [29], and USA [30]. Besides *Foc*, other Fusarium species, including *F. solani* [19], *F. verticillioides* [31], *F. equiseti* [18], and *F. clavus* [31], have also been reported to be associated with pepper Fusarium wilt. Similarly, the causal agents of potato Fusarium wilt also include several Fusarium species as well according to the disease reported in different countries with various latitudes and growing conditions [32]. It was first reported in the USA in 1924 [33] and has been reported worldwide [34–40]. *F. oxysporum* f.sp. *tuberosi* (*Fot*) was reported to be the most dominant agent of potato Fusarium wilt [34]. In addition, *F. solani*, *F. sambucinum* [34, 41], *F. equiseti*, *F. redolens*, *F. acuminatum* [34], *F. brachygibbosum* [37], *F. foetens* [42], and *F. chlamydosporum* [29] have also been reported to cause the disease independently. Eggplant Fusarium wilt was first reported in 1958 in Japan [43] and has been reported to occur worldwide. The causal agents of eggplant Fusarium wilt are associated with a taxonomically diverse set of Fusarium species, including *F. oxysporum* [43, 44], *F. solani* [45], *F. chlamydosporum* [29], *F. elaeidis* [46], *F. falciforme* [47], *F. equiseti* [48], *F. incarnatum*, and *F. proliferatum* [45]. However, this disease is caused by a single Fusarium species without co-infection [45]. Among the reported agents, *F. oxysporum* f. sp. *melongenae* (*Fom*) remains the most frequently documented and economically important agent of eggplant wilt worldwide [24, 43, 45, 49, 50]. Tomato Fusarium wilt is predominantly caused worldwide by *F. oxysporum* f. sp. *lycopersici* (*Fol*), which is currently classified into three races based on their ability to overcome specific host resistance genes [51]. However, few studies have reported that other *Fusarium* species also can cause wilt symptoms on tomato in different geographical regions, such as *F. solani* in India [52, 53] and *F. brachygibbosum* in China [54].

## Current status of genome sequences of *Fo* infecting solanaceous vegetables

### Genomes of *Foc* and *Fom* without chromosome-level assemblies

The first whole genome sequence of *Foc* isolate 14 003 that was originally collected from China was generated using second-generation Illumina sequencing technology. The resulting assembly spans 47.6 Mb and comprises 796 contigs with an N50 value of 209 562 bp and an average genome coverage 59.0× [55]. The annotated genome contains 15 762 predicted protein-coding genes, and the overall GC content was 51% (Table 1). Next, a draft genome sequence of another *Foc* isolate pep1 from China was generated using third-generation and long-read Oxford Nanopore sequencing technology, which produced an assembly containing 34 contigs with a genome size of 54.5 Mb [27] . The contig N50 increased substantially to 4 962 668 bp compared to the previous assembly, while the GC content was estimated at 47.6% (Table 1). The first genome sequence of an *Fom* isolate 14 004, collected from China, was generated using Illumina sequencing technology, which produced an assembly comprising 1631 scaffolds with a total length of 54 Mb and an N50 value of 56 828 bp [56]. The GC content of this assembly is 46.4% and 16 485 protein-coding genes were predicted (Table 1). In contrast, an optical map of a *Fom* isolate 109, collected from Turkey, was generated using Bionano Genomics (BNG) technology and yielded an estimated genome size of 57.7 Mb spanning 15 chromosomes with an optical map N50 of 850 000 bp, in the absence of genome sequencing [57]. Notably, the genome assembly of *Fom* 14 004 does not fully align with the optical map of *Fom* 109, suggesting the presence of unsequenced genomic regions in *Fom* [57]. Currently, no genome information for *Fot* has been reported. Collectively, chromosome-level genome assemblies for *Foc* and *Fom* based on sequencing data remain unavailable, likely due to the high abundance of repetitive and transposable elements in both genomes.

**Table 1:** Summary of genome assembly and sequence information of *Fo* infecting solanaceous vegetables.

**Name**	**Host**	**Sequencing technique**	**Sequence coverage (×)**	**Chromosome Number**	**Contig number**	**Contig N50 (bp)**	**Scaffold Number**	**Scaffold N50 (bp)**	**GC content (%)**	**Total length (bp)**	**GenBank Accession number**
*Foc*pep1 [27]	*C. annuum*	Oxford Nanopore	200	-	34	4,962,668	34	4,962,668	47.6	54,516,562	GCA_016801315.1
*Foc*14003 [55]	*C. annuum*	Illumina	59	-	1,154	98,506	739	209,562	51.0	47,541,829	GCA_014770115.1
*Fom*14004 [56]	*S. melongena*	Illumina	59	-	2,086	373,752	1,631	585,845	46.4	53,986,354	GCA_001888865.1
*Fol*4287 [58]	*S. lycopersicum*	Sanger+optical map	6.28+55	15	1,362	95,416	114	1,976,106	48.5	61,386,934	GCA_000149955.2
*Fol*007	*S. lycopersicum*	Illumina	88	-	3,280	118,633	1,999	137,832	48.0	51,322,760	GCA_001702905.1
*Fol*MN25	*S. lycopersicum*	Illumina	210	-	801	435,126	388	2,091,760	48.0	48,637,398	GCA_000259975.2
*Fol*4287 [60]	*S. lycopersicum*	PacBio+Illumina	10+66	13	503	1,338,693	498	1,338,693	47.5	53,866,543	GCA_003315725.1
*Fol*4287 [61]	*S. lycopersicum*	PacBio RSII	73	-	47	4,140,197	47	4,140,197	48.0	56,213,053	GCA_001703175.2
*Fol*001 [62]	*S. lycopersicum*	PacBio RSII	58	-	87	3,629,953	87	3,629,953	47.5	55,816,077	GCA_015345885.1
*Fol*010 [62]	*S. lycopersicum*	PacBio RSII	67.66	-	53	4,612,997	53	4,612,997	47.5	54,813,233	GCA_015345895.1
*Fol*59 [62]	*S. lycopersicum*	Oxford Nanopore	75.5	-	243	3,035,620	243	3,035,620	48.0	53,285,344	GCA_021018635.1
*Fol*D11 [62]	*S. lycopersicum*	PacBio RSII	99.8	14	38	4,408,267	38	4,408,267	48.0	57,229,625	GCA_003977725.1
*Fol*007 [63]	*S. lycopersicum*	Oxford Nanopore+Illumina+Hi-C	123.51+89.68+103.73	15	35	4,175,218	35	4,176,489	47.8	56,041,038	-

### Reference genome of *Fol* based on first-generation sequencing technology

To date, more than thirty assembled genomes of *Fol* have been deposited in NCBI. However, the majority of these genomes were generated using second-generation sequencing technology, resulting in a limited number of chromosome-level genome assemblies. The only reference genome of *Fol* is the chromosome-level assembly of the *Fol*4287 genome, which was sequenced in 2010 using the first-generation Sanger sequencing technology at a sequencing depth of 6.28× [58]. The assembled *Fol*4287 genome spans approximately 61.4 Mb and consists of 1 362 contigs, which were assembled and anchored to 15 chromosomes with the assistance of a 55× coverage optical map. The genome contains 28% repetitive sequences (Table 1). Genome annotation of *Fol*4287 produced 17 735 predicted protein-coding genes, and comparative analysis with the closely related Fusarium species *F. verticillioides* identified *Fol* lineage-specific genomic regions (LS regions), including chromosomes 3, 6, 14, and 15, which are enriched in transposons and repetitive sequences and contain one large intra-chromosomal and two inter-chromosomal segmental duplications within Chr3 and Chr6 [58]. Inevitably, this reference genome contains sequencing and annotation errors due to the relatively low sequencing coverage and technological limitations of the Sanger-based approach, as well as the absence of transcriptomic data to support gene predictions [59]. For example, several known Secreted In Xylem (SIX) effector genes, including *SIX7*, *SIX8*, *SIX11*, *SIX12*, and *SIX14*, were not originally annotated in the *Fol*4287 genome, while *SIX10* possesses annotation errors, which hinders the comprehensive identification of novel effectors, thereby limiting insights into the molecular basis of *Fol* virulence [59]. Besides *Fol*4287, genome sequences of Race 2 strain *Fol*007 and Race 3 strain *Fol*MN25 based on second-generation Illumina sequencing have been generated, but without high-quality assemblies (Table 1). These findings indicate that *Fol* genome assemblies generated using both the first-generation Sanger sequencing and second-generation Illumina sequencing platforms are constrained by sequencing, assembly, and annotation inaccuracies, underscoring the need for more complete and high-fidelity genomic resources.

### *Fol* genome sequences based on third-generation sequencing technologies

Following the leap of sequencing technology, especially the emergence of low-cost and long-read third-generation sequencing platforms, such as Oxford Nanopore and PacBio, several updated genome sequences of *Fol*4287 have been either reported or deposited in the NCBI database. The first updated assembly of the *Fol*4287 genome was released in 2018, and was generated using 66× coverage of Illumina and 10× coverage of PacBio sequencing technologies. This version resulted in a 53.9 Mb assembly consisting of 499 contigs. However, this assembly did not achieve chromosome-level resolution and lacked functional genome annotations (Table 1) [60]. Subsequently, in 2020, the *Fol*4287 genome was resequenced using a high-coverage PacBio third-generation sequencing platform, but also failed to reach chromosome-level completeness and was not accompanied by detailed genome annotation (Table 1) [61]. Notably, this assembly revealed that contig 14 corresponds to the pathogenic chromosome Chr14 identified in the 2010 assembly, which contains all *SIX* genes except for *SIX8*. Interestingly, contig 58, containing *SIX8*, overlaps with contig 14, suggesting that all *SIX* genes are likely located on a single pathogenic accessory chromosome [61]. Following these updates, several additional *Fol* genomes have been sequenced using high-coverage third-generation sequencing technologies, including Race 1 strains (*Fol*001, *Fol*010), the Race 2 strain (*Fol*59), and the Race 3 strain (*Fol*D11), but their assemblies still fall short of chromosome-level quality [62]. Collectively, these studies highlight that third-generation sequencing technologies greatly surpass second-generation platforms in assembly quality, enabling the development of high-quality, chromosome-level reference genomes for *Fol*.

### Chromosome-level assembly of *Fol*007 as a new reference genome using third-generation sequencing technologies

Recently, the genome of race 2 strain *Fol*007 was sequenced and assembled at the chromosome level using third-generation Nanopore long-read sequencing with 123.51× coverage in combination with high-throughput chromosome conformation capture (Hi-C) data at 103.73× coverage [63] (Table 1). This assembly comprises 15 chromosomes and 25 telomeres, including 10 complete chromosomes, and encodes 29 148 proteins, encompassing all known *SIX* genes except *SIX4*, which is not present in *Fol* race 2. Compared with *F. verticillioides*, the *Fol*007 genome harbors four complete lineage-specific (LS) chromosomes, including Chr5, Chr13, Chr14, and Chr15. Among these, Chr13 harboring all known *SIX* genes (except *SIX4* and *SIX8*) and three novel candidate effector genes corresponds to the pathogenicity chromosome Chr14 in *Fol*4287, and remains stable across different *Fol* strains. Comparative genomic analysis identified a telomeric duplication of an LS region containing *SIX8* and *PSL1* on LS chromosome 15 and core chromosomes 6, 7, and 11. In contrast, segmental duplications were absent on LS chromosomes in the *Fol*007 genome [63]. Additionally, an active and *Fol*-specific *Foxy* transposable element that inactivated a second copy of *SIX13* was identified. This element has expanded in *Fol* race 2 and 3 genomes compared with those of race 1 and non-pathogenic strains, potentially contributing to the evolutionary diversification of *F. oxysporum* [63]. Importantly, the *Foxy* element may serve as a potential genetic marker for phylogenetic analyses of *F. oxysporum* formae speciales. Collectively, this high-quality genome assembly provides a robust reference for studying genome structural variations, evolutionary dynamics, and virulence mechanisms in *Fol*.

## Multifaceted virulence mechanisms utilized by *Fo* to infect solanaceous vegetables

### Regulation of conidiation and conidial germination during *Fol* infection

**Figure 1:**
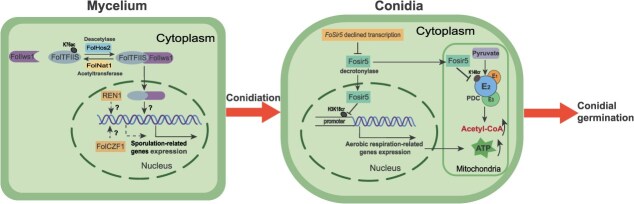
Regulatory mechanisms controlling conidiation and conidial germination during *Fol* infection. Conidiation in *Fol* is governed by transcriptional regulators, including REN1 and FolCZF1, and by post-translational control of FolTFIIS. The deacetylase FolHos2 removes acetyl groups from FolTFIIS, enabling its interaction with FolIws1 and subsequent nuclear import, where it activates the transcription of sporulation-related genes to promote microconidia, macroconidia, and chlamydospore formation. Conidial germination is negatively regulated by the mitochondrial decrotonylase FoSir5, which interacts with the E2 subunit of PDC to remove crotonyl modifications. In the nucleus, FoSir5 decrotonylates histone H3K18 to inhibit the expression of sporulation-related genes. During germination, the decreased expression of *FoSir5* leads to increased PDC activity and enhanced transcription of aerobic respiration-related genes, thereby enhancing acetyl-CoA production and ATP generation to promote germination.

*Fol* initiates infection of tomato roots through the germination of resting chlamydospores, which is triggered by root exudates of tomato plants in the soil. Hyphae emerging from chlamydospores have the ability to penetrate the root epidermis either through natural openings or wounds [9]. Following root penetration, *Fol* colonizes the root cortex and reaches the xylem vessels, where it propagates by producing microconidia that spread throughout the vascular system. These microconidia germinate to form an abundant hyphal network, which can physically block the xylem vessels, thereby impeding water transport and leading to the characteristic wilting symptoms associated with the disease [9]. Therefore, the coordinated process of microconidia production and germination is an indispensable step for successful infection and colonization by *Fol*. The regulation of conidiation in *Fol* involves several key transcriptional regulators. The transcriptional regulator *REN1*, encoding a protein homologous to MedA of *Aspergillus nidulans* and Acr1 of *Magnaporthe grisea*, is involved in the development of microconidia and macroconidia, but not chlamydospores, in *F. oxysporum* [64]. Similarly, *FolCZF1*, encoding a C_2_H_2_ transcription factor, is required for conidiation in *Fol* (Fig. 1) [65]. Recent studies have highlighted the key role of post-translational regulation in this process. Specifically, the deacetylation of transcription elongation factor IIS (TFIIS), which is highly conserved in a wide range of eukaryotic organisms, by the FolHos2 deacetylase is indispensable for the process of conidiation [66]. Following deacetylation, FolTFIIS is translocated into the nucleus through its direct interaction with FolIws1, a protein containing a nuclear localization signal. This nuclear translocation activates the transcription of sporulation-related genes, ultimately promoting the production of microconidia, macroconidia, and chlamydospores (Fig. 1) [66].

Recent studies have advanced our understanding of the molecular mechanisms that regulate conidial germination in *F. oxysporum*. One key factor identified is FoSir5, which acts as a negative regulator of this process [67]. FoSir5 possesses decrotonylase enzymatic activity and interacts with the E2 component dihydrolipoyllysine acetyltransferase (FoDLAT) of the pyruvate dehydrogenase complex (PDC) within mitochondria. Through the removal of crotonyl modifications from FoDLAT, FoSir5 attenuates PDC activity and acetyl-CoA production, which in turn leads to a consequent reduction in the cellular energy supply, thereby inhibiting conidial germination. Meanwhile, in the nucleus, FoSir5 decrotonylates histone H3K18, resulting in reduced expression of aerobic respiration-related genes, thereby repressing ATP generation. In contrast, downregulation of *FoSir5* increases ATP production, providing the energy required for germination and facilitating conidial germination [67] (Fig. 1). Collectively, these findings demonstrate that both the formation and germination of microconidia are indispensable for the successful infection process of *Fol*. This developmental program is tightly regulated by multiple molecular factors, including the transcriptional regulators REN1, FolCZF1, and deacetylated FolTFIIS, which coordinate conidial development, as well as the mitochondrial and nuclear enzyme modulator FoSir5, which governs the metabolic control of conidial germination. Together, these regulators form an integrated network that ensures efficient sporulation, energy adaptation, and host colonization during *Fol* pathogenesis.

### Regulation of effector gene expression in *Fol*

**Figure 2:**
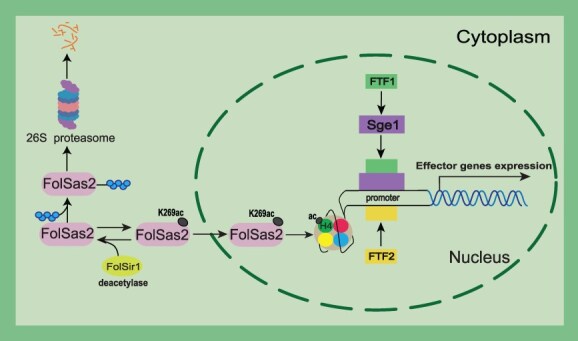
Regulatory mechanisms controlling effector gene expression during *Fol* infection. Transcription of effector genes is controlled by chromatin modification and a network of transcription factors. During the early infection stage, increased levels of histone acetyltransferase FolSas2 undergoes autoacetylation at lysine 269, which prevents its 26S proteasome-mediated degradation and enables histone H4K8 hyperacetylation, promoting transcription of effector genes. At other stages, elevated FolSir1 deacetylates FolSas2, promoting FolSas2 degradation. The WOPR-domain-containing transcription factor Sge1 directly activates *SIX* and other effector genes, and the pathogenicity chromosome-encoded FTF1, a homolog of Sge1, enhances effector gene expression in an Sge1-dependent manner during infection. Its core-chromosome homolog FTF2 also induces effector gene expression by binding to their promoters.

The expression of *Fol* effector genes is tightly controlled by a complex regulatory network involving both transcriptional and post-translational mechanisms (Fig. 2). During early infection, autoacetylation of FolSas2, a histone acetyltransferase, at lysine 269 prevents its degradation by the 26S proteasome and promotes histone H4K8 hyperacetylation, thereby promoting the activation of effector genes required for pathogenicity [68]. In contrast, deacetylation of FolSas2 by the deacetylase FolSir1 reduces its stability and results in impaired pathogenicity [68]. The transcription factor Sge1 (*SIX* gene expression 1), containing a WOPR-type DNA-binding domain, is required for *Fol* pathogenicity and lifestyle switches and can directly activate the expression of effector genes, such as the *SIX* genes [69]. Similarly, *FTF1*, a transcription factor located on the pathogenicity chromosome and homologous to *Sge1*, is upregulated during infection and activates effector genes expression in an Sge1-dependent manner [70]. In addition, the *FTF2* transcription factor, encoded by a gene located on the core chromosomes and homologous to *FTF1*, induces the expression of effector genes by directly binding to their promoters. Both regulatory activities contribute to *Fol* virulence, as evidenced by RNA silencing of *FTF1* and *FTF2*, which leads to reduced virulence and downregulation of effector genes, such as *SIX1* and *SIX6* [70].

### SIX effectors in the xylem battleground

*Fol* secretes a variety of molecules, including secondary metabolites, cell wall-degrading enzymes, and effectors, to facilitate host colonization and disease progression [12]. Among these, effectors are considered key determinants of *Fol* pathogenicity [13]. To date, 14 SIX proteins have been identified using proteomic analysis of xylem sap isolated from infected tomato plants. These SIX effectors are typically small and cysteine-rich proteins, with cysteine residues forming disulfide bonds that enhance protein stability [13, 71–73].

**Figure 3:**
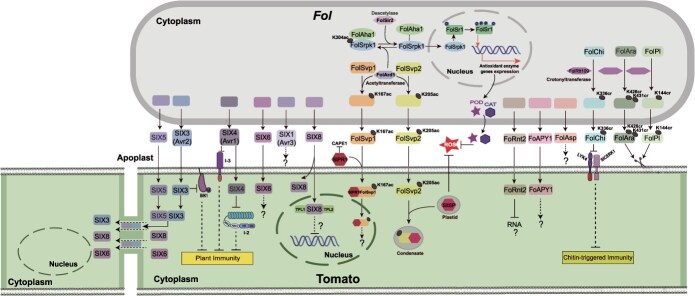
A schematic illustration summarizing diverse mechanisms by which *Fol* effectors manipulate tomato immunity. *Fol* effectors manipulate immune signaling, transcriptional regulation, ROS homeostasis, and cell-to-cell communication to facilitate its colonization. SIX4/Avr1 suppresses I-2- and I-3-mediated resistance. The SIX3/SIX5 complex localizes to plasmodesmata and enables intercellular trafficking of SIX3/Avr2, SIX6, and SIX8; Avr2 additionally suppresses immunity by disrupting BIK1 mono-ubiquitination. SIX6 acts in both the cytoplasm and nucleus, and SIX8 targets nuclear TOPLESS co-repressors (TPL1 and TPL2) to repress defense gene expression. *Fol* also secretes acetylation-dependent virulence factors: acetylated FolSvp1 binds apoplastic SlPR1 to block CAPE1 generation, whereas acetylated FolSvp2 promotes the translocation of the tomato iron–sulfur protein SlISP from plastids into effector condensates, thereby suppressing ROS production. In response to host-derived ROS, deacetylated FolSrpk1 enters the *Fol* nucleus and activates antioxidant enzyme genes via phosphorylating FolSr1, thereby promoting the secretion of POD and CAT enzymes to degrade ROS. Crotonyltransferase FolRtt109 catalyzes chitinase FolChi at lysine 336, arabinofuranosidase FolAra at lysine 426 and 431, and pectate lyase FolPl at lysine 144 to suppress chitin-triggered immunity and promote host cell-wall degradation. Additional secreted effector proteins, including the ribonuclease FoRnt2, peptidase FoAPY1, and aspartic protease FolAsp, further promote infection by degrading host RNA, proteins, and likely suppressing pattern-triggered immunity.

**Figure 4:**
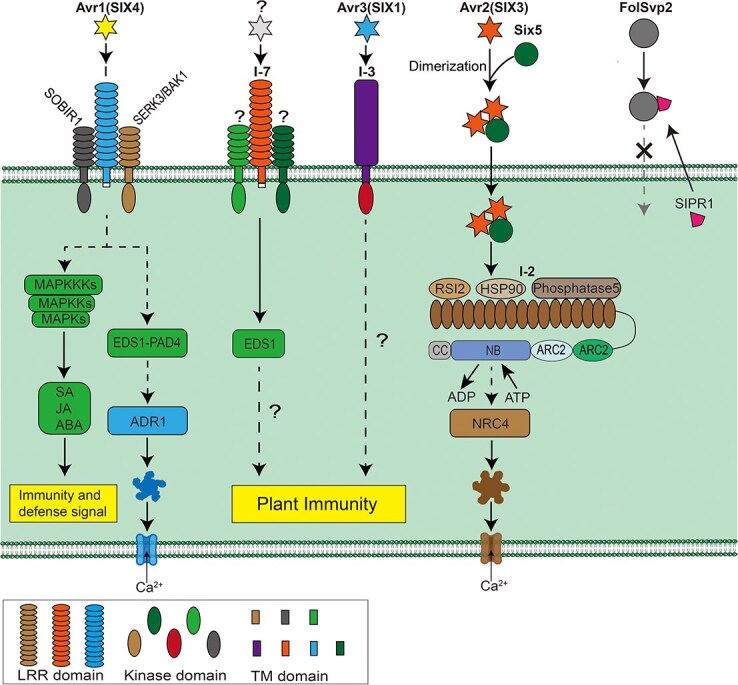
Diverse immune receptors and signaling networks mediating tomato resistance to *Fol*. This model shows that the cultivated tomato employs cell-surface and intracellular receptors to recognize *Fol* avirulence effectors and activate their unclear downstream PTI/ETI signaling components, such as Ca^2+^ influx, ROS production and MAPK activation, and JA/SA/ABA/ET crosstalk, which fine-tunes defense outputs and restricts *Fol* colonization. The LRR receptor-like protein I perceives Avr1 and activates signaling via the co-receptors SOBIR1 and SERK3/BAK1, likely leading to the activation of MAPK cascades and the EDS1-PAD4 hub, which likely cooperates with the helper NLR ADR1 and hormonal pathways involving SA, JA, and ABA to drive Ca^2+^ influx and defense gene expression. Another LRR-RLP, I-7 from *S. pennellii*, confers broad resistance to multiple *Fol* races in an EDS1-dependent manner, although its cognate effector remains unknown. The S-domain receptor-like kinase I-3 mediates specific recognition of Avr3 in the apoplastic space. Inside the cell, the CC-NB-LRR protein I-2 recognizes Avr2 together with its partner SIX5, which depends on HSP90, RSI2, and phosphatase 5 for receptor stabilization and activation, ultimately signaling through the helper NLR NRC4 to induce Ca^2+^ influx and robust effector-triggered immunity. In parallel, the apoplastic SlPR1 secreted by tomato blocks the entry of the acetylation-dependent virulence effector FolSvp2 into host cells, thereby preventing FolSvp2 from further manipulation of host cells and conferring resistance against *Fol*.

SIX1/Avr3 plays dual roles in virulence and avirulence. SIX1 is a ~32-kDa secreted protein consisting of an N-terminal signal peptide for secretion, a cleaved prodomain, and a mature domain containing eight conserved cysteines that form disulfide bonds for structural stability within the xylem sap [72–74]. Functional analyses have demonstrated that SIX1 is indispensable for full virulence, as *SIX1* knockout mutants exhibit reduced disease symptoms, while complementation restores pathogenicity [71]. In addition, SIX1 is genetically recognized by the tomato resistance gene *I-3*, which encodes an extracellular S-like receptor kinase (RLK) [71]. Therefore, SIX1 is designated as Avr3 [71]. This recognition between Avr3 and I-3 likely occurs in the apoplast and may involve extracellular plant targets, consistent with the secretion of SIX1 into the xylem (Fig. 4) [75]. The molecular mechanism by which SIX1 promotes virulence remains incompletely understood (Fig. 3). Gene expression analyses reveal that *SIX1* is strongly induced during host infection, including in interactions with isolated plant cells, which suggests that the induction is independent of the whole-plant context or specific root morphology, thereby implying that a host cell-derived signal serves as the activator to induce its expression [76]. *SIX1* homologs are highly conserved across multiple *F. oxysporum* formae speciales, but their number, sequence variants, and functional specificity exhibit variability. For example, *F. oxysporum* f. sp. *cubense* (*Foc*) Tropical Race 4 contains three paralogs of *SIX1*, including *SIX1a*, *SIX1b*, and *SIX1c*, but only *SIX1a* is required for full virulence on Cavendish banana, with complementation of the *ΔSIX1a* mutant restoring pathogenicity [77, 78]. In addition, *SIX1a* homologs are found in other *Foc* races, suggesting they originate from an ancient and conserved evolutionary lineage [78]. A homolog of *SIX1* was also identified in *F. oxysporum* f. sp. *conglutinans* (*Focn*), and the *ΔFocn-SIX1* mutant displayed reduced pathogenicity, indicating that *Focn-SIX1* is required for full pathogenicity on kale [78]. A *SIX1* homolog, sharing 81.34% sequence similarity, was identified in the *Fot* isolate FPo, which was isolated from potato showing Fusarium wilt symptoms in India, but its role in *Fot* virulence remains unknown [54]. Interestingly, the pathogenic role of *Focn-SIX1* appears to be host-specific, as *Fol-SIX1* cannot complement the function of *Foc-SIX1* on kale [78].

SIX3 is Avr2 and the SIX3/SIX5 pair promotes cell-to-cell movement of effectors. The protein encoded by the *SIX3* gene of *Fol* also contains an N-terminal signal peptide, similar to SIX1, but differs from most other SIX proteins by possessing only two cysteine residues [72]. The *SIX5* gene, positioned upstream of *SIX3,* shares a bidirectional promoter region with *SIX3*, and encodes a 12 kDa protein containing six cysteine residues [72, 73]. SIX3 can form a homodimer or interact with SIX5 to form a heterodimer complex [79, 80]. SIX3 is recognized by the tomato resistance protein I-2 within plant cells to trigger an immune response against *Fol*; therefore, SIX3 is named Avr2 [81]. The interaction between Avr2 and SIX5 occurs at plasmodesmata, enabling cell-to-cell movement of Avr2, SIX6, and SIX8, which enhances virulence in susceptible tomato plants (Fig. 3) [82, 83]. In addition, Avr2 and BOTRYTIS-INDUCED KINASE 1 (BIK1) co-localize *in planta*, compromising BIK1 mono-ubiquitination, thereby reducing its abundance and altering its localization to the plasma membrane, ultimately suppressing immune signaling (Fig. 3) [84]. Avr2 exhibits structural similarity to SIX7 and SIX8, placing all three within the ToxA-like structural family [85, 86].

SIX4/Avr1 suppresses R-gene-mediated resistance. SIX4 is recognized by the tomato resistance protein I, and is therefore designated as Avr1 [87]. Although deletion of *SIX4* does not reduce the virulence of *Fol* in susceptible tomato plants, Avr1/SIX4 suppresses CC-NB-LRR protein I-2- and the S-like receptor kinase (SRLK) I-3-mediated resistance [87], while resistance mediated by LRR receptor-like protein (LRR-RP) remains unaffected (Fig. 3) [75, 88]. Although *Fol-SIX4* is not directly required for the full virulence of *Fol* on susceptible tomato plants, the homolog of *SIX4* in *F. oxysporum* (*Fo*) isolate *Fo*5176 infecting *Arabidopsis* contributes to *Fo*5176 virulence [74]. Notably, a *SIX4* homolog in *F. oxysporum* f. sp. *conglutinans* (*Foc*), the causal agent of cabbage wilt disease, shares 99% nucleotide identity with *Fol-SIX4* and the knockout of *Foc-SIX4* in *Foc* results in reduced virulence, confirming its role in virulence rather than as an avirulence determinant [89, 90].

SIX6 is conserved in Fusarium and displays diverse roles in infection and resistance. The effector SIX6, a 225 amino acid protein, is also secreted into the xylem of tomato during infection by *Fol* [72]. Expression of *SIX6* occurs both at early and late stages of tomato infection with the presence of a living host, indicating that host-derived signals are required for its activation [91]. Moreover, GFP-tagged SIX6 localizes to both the nucleus and cytoplasm of plant cells (Fig. 3) [91]. *Fol* Δ*SIX6* mutants exhibit significantly reduced disease symptoms, as evidenced by reduced disease index and increased plant biomass compared with wild-type strains [91]. In soybean, the presence of the *SIX6* effector gene is positively correlated with wilt symptom severity [92]. Similarly, *SIX6* contributes to the virulence of *F. oxysporum* f. sp. *niveum* (*Fon*) in watermelon [93]. In addition, *F. oxysporum* f. sp. *radicis-cucumerinum* (*Forc*) relies on SIX6 for efficient colonization and symptom development in cucurbits [94]. Despite these conserved features, SIX6 is not universally required for virulence. In *F. oxysporum* f. sp. *melonis* (*Fom*), deletion of *SIX6* does not affect pathogenicity on melon plants, as *Fom* Δ*SIX6* mutants induce wilt symptoms comparable to those caused by the wild type [95]. This implies that SIX6 is dispensable in *Fom* or that functional redundancy exists among effectors. In tomato, further studies revealed that deletion of a major region of the *Fol* pathogenicity chromosome, carrying *SIX6* as well as *SIX9* and *SIX11*, surprisingly, does not impair virulence on tomato [61]. These findings suggest that *Fol* pathogenicity chromosomes likely contain a network of functionally redundant virulence effectors, where the loss of a single effector can be masked by the activity of others. Recently, SIX6 was identified as an avirulence gene *AvrFW1*, which triggers FW1-mediated resistance in *F. oxysporum* f. sp. *fragariae* (*Fof*) race 1 during strawberry infection, as deletion of *SIX6* enabled virulence on FW1-carrying strawberry cultivars [96]. In addition, SIX6 has been recognized as a structurally distinct member of the FOLD effector family [86] .

SIX8 targets TOPLESS to suppress plant immunity. The xylem sap of tomato plants infected with *Fol* contains another 13 kDa protein designated SIX8 [73]. Unlike other *SIX* genes, *SIX8* occurs in multiple copies [73], leading to difficulties in generating *SIX8* knockout mutants. Nine identical copies of *SIX8* were initially identified in the *Fol*4287 genome assembled using Sanger sequencing, whereas in the updated *Fol*4287_2020 genome assembly based on PacBio sequencing, eight copies of *SIX8* were annotated [59, 97]. In contrast, the *Fol*007 genome assembly based on Nanopore long-read sequencing contains four copies of *SIX8* [63]. The presence of multiple copies of *SIX8* in the *Fol* genome has hindered the precise determination of its contribution to virulence. Recent studies, however, revealed that SIX8 specifically interacts with two members of the tomato TOPLESS co-repressor family, TPL1 and TPL2 (Fig. 3) [98]. Loss-of-function mutations in *TPL1* strongly reduced susceptibility to *Fol,* and the *tpl1*:*tpl2* double mutant exhibited an even higher level of resistance, demonstrating that SIX8 targets and manipulates tomato TOPLESS proteins to promote *Fol* infection [98]. The *Fol*4287 genome also harbors a homologous gene of *SIX8*, designated *SIX8b* [73]. While *SIX8b* is present in the genome, it has not been detected in the xylem sap of infected tomato plants, indicating that its expression is below detectable levels or absent during *Fol* infection [73]. In addition, homologs of the *SIX8* gene have been identified in the genomes of other *F. oxysporum* formae speciales, including *F. oxysporum* f. sp. *cubense* (*Foc*), causing banana wilt, *F. oxysporum* f. sp. *spinaciae* infecting spinach, and *F. oxysporum* f. sp. *lactucae* (*Fola*) causing Fusarium wilt of lettuce [99–101]. In *Foc* tropical race 4 (FocTR4), *SIX8* knockout mutants displayed reduced virulence on Cavendish banana [102].

### Acetylation-dependent virulence factors promote *Fol* virulence

Comprehensive acetylome analysis of *Fol* secretome identified 50 acetylated secreted proteins, among which 32 novel candidate genes are located on core chromosomes rather than LS chromosomes. Many of these candidates are up-regulated during early infection and have been experimentally validated as novel pathogenicity determinants [103]. The secreted effectors FolSvp1 and FolSvp2 exemplify how acetylation fine-tunes *Fol* virulence (Fig. 3). FolSvp1 is acetylated at lysine 167 by the acetyltransferase FolArd1, which prevents ubiquitination-mediated degradation and thereby stabilizes the protein. The stabilized FolSvp1 directly interacts with the tomato pathogenesis-related protein 1, SlPR1 [104]. This interaction mediates the translocation of SlPR1 from the apoplast into the host nucleus, thereby abolishing CAPE1 peptide production and facilitating *Fol* infection [104]. Similarly, FolSvp2 depends on acetylation at lysine 205 to prevent ubiquitination-mediated degradation in both *Fol* and host cells. Acetylated FolSvp2 promotes the translocation of the tomato iron–sulfur protein SlISP from plastids into effector condensates *in planta*, thereby suppressing reactive oxygen species (ROS) production and promoting colonization [105]. Interestingly, tomato plants have evolved a counter-defense strategy in which apoplastic SlPR1 sequesters FolSvp2, blocking its entry into host cells and abolishing its virulence activity [105]. This discovery highlights an unanticipated role of PR1 as an active component of plant immune defense (Fig. 4). Beyond these effectors, deacetylation of FolSrpk1 at lysine 304 contributes to *Fol* infection [106]. Upon ROS exposure, FolSrpk1, deacetylated by the deacetylase FolSir2, dissociates from its cytoplasmic chaperone FolAha1 and translocates to the nucleus, where it hyperphosphorylates its target FolSr1. This modification activates the transcription of antioxidant enzyme genes, leading to the secretion of enzymes that detoxify host-derived hydrogen peroxide (H₂O₂), including peroxidase (POD) and catalase (CAT), ultimately promoting successful *Fol* colonization and virulence [106].

### Enzymatic effectors require lysine crotonylation to facilitate *Fol* infection

During infection, *Fol* secretes enzymatic effectors, such as chitinase and plant cell wall-degrading enzymes (CWDEs), to degrade plant cells and promote entry into plant cells. A recent study showed that the fungal-specific conserved crotonyltransferase, FolRtt109, catalyzes lysine crotonylation on several enzymatic effectors, including chitinase FolChi at lysine 336, arabinofuranosidase FolAra at lysine 426 and 431, and pectate lyase FolPl at lysine 144, thereby enhancing their enzyme activity required for *Fol* infection [107]. Increased FolChi activity can suppress chitin-triggered immunity, while FolAra and FolPl promote host cell-wall degradation (Fig. 3). Interestingly, chemical inhibition of FolRtt109 blocks the crotonylation of these enzymes, resulting in reduced infection by *Fol*. In addition, this chemical inhibitor also reduces the infection of other economically important fungal pathogens, such as *Botrytis cinerea* causing gray mold in bean leaves, *F. graminearum* responsible for Fusarium head blight in wheat, and *Magnaporthe oryzae* causing rice blast, without affecting the growth of the host plants. These findings suggest the molecular mechanisms employed by *Fol* are likely conserved across fungal pathogens, including Fusarium species, highlighting the potential of chemical inhibitors as broad-spectrum agents against fungal infections. Future studies could examine whether this chemical inhibitor reduces infection by *Foc*, *Fom*, and *Fot*.

### Other effectors with diverse strategies contribute to *Fol* virulence

In addition to the SIX and acetylation-dependent virulence factors, a range of other secreted proteins have been reported to contribute to *Fol* pathogenicity (Fig. 3). For example, the secreted ribonuclease T2 protein FoRnt2 promotes *Fol* virulence through the degradation of host RNA [108]. The secreted peptidase FoAPY1 exhibits peptidase activity *in vitro*, and the knockout of *FoAPY1* significantly reduced *Fol* virulence on tomato plants, whereas its overexpression in tomato seedlings enhances susceptibility to both *F. oxysporum* and *B. cinerea*, demonstrating that FoAPY1 likely contributes to virulence through peptidase activity against host proteins [109]. Similarly, the aspartic protease FolAsp is specifically induced in the host root during infection [110]. *FolAsp* knockout mutants exhibit markedly reduced virulence, whereas overexpression enhances pathogenicity. Functional assays further show that *Fol*Asp inhibits INF1-induced cell death and flg22-triggered ROS burst *in planta* [110]. Collectively, these findings underscore the diverse molecular strategies employed by *Fol* to modulate host immunity and promote infection. However, further research is needed to elucidate the precise molecular mechanisms underlying the functions of these non-SIX effectors.

### The effector study in *Fom* is still in its early stages

Using a bioinformatic pipeline with the total protein of *Fom* as input, a total of 1019 proteins with signal peptides, but without transmembrane domains and glycosylphosphatidylinositol (GPI) anchors, were predicted as effector candidates [111]. Among these, 194 effector candidates were further identified based on an amino acid length of fewer than 300 residues. Through label-free quantitative proteomics analysis of secreted proteins during *Fom* infection of eggplant, 42 effector candidates were further selected for functional analysis. Functional analysis revealed that among the 42 candidates, those examined candidates contained functional signal peptides, and CSEP g3195 was shown to suppress BAX-triggered programmed cell death in *N. benthamiana* [111], suggesting that CSEP g3195 likely contributes to *Fom* pathogenicity by suppressing the host defense response. Despite the identification of effector candidates in *Fom*, their role during the infection of eggplant remains unclear. Future studies should focus on understanding the virulence role of these effectors and determining whether they employ similar strategies of *Fol* effectors in facilitating *Fom* infection in eggplant.

## Host resistance against *Fo* infection in solanaceous vegetables

### Resistance loci and candidate genes in eggplant and its relatives that confer resistance to *Fom*

Resistance to *Fom* has been identified in cultivated eggplant (*S. melongena* L.), as well as in its wild and allied relatives, and resistance loci and QTLs have also been mapped. Several eggplant germplasms, including LS174, LS1934, and LS2436, were reported to harbor a single dominant resistance locus that confers full resistance to *Fom* [112]. Physical mapping using these resistance lines identified a major allelic locus, *FM1*, at the end of chromosome 2, with *Fm1^L^* detected in LS1934 and *Fm1^E^* detected in EPL-1 cultivar derived from LS174, whereas resistance from LS2436 mapped to a distinct locus in the mid-region of chromosome 4 [112, 113]. Several eggplant-related species, including *S. aethiopicum* [114], *S. torvum* [115], *S. incanum* [48], *S. violaceum*, and *S. sisymbriifolium* Lam [116], have been reported to exhibit resistance to *Fom* infection. Therefore, interspecific hybrid eggplant lines were generated by introgressing resistance loci from species such as *S. aethiopicum* [114], *S. indicum*, *S. integrifolium* [116], and *S. macrocarpon* [117] into cultivated eggplant. A single dominant resistance locus *Rfo-sa1* (Resistance to *F. oxysporum* f. sp. *melongenae* from *S. aethiopicum* 1) was identified in the somatic hybrid line 305E40, introgressed from *S. aethiopicum*. This locus was mapped to chromosome 2 of eggplant, closely linked to and orthologous to the *FM1* locus [112] [118, 119]. In addition, two quantitative trait loci (QTLs), *FomE02.01* on chromosome 2 and *FomE11.01* on chromosome 11 of eggplant, associated with resistance to *Fom*, were identified from the F2 population of 305E40 [120]. Recently, QTL *FomCH02* on chromosome 2 that provides full resistance to *Fom*, and QTL *FomCH11* on chromosome 11 conferring partial resistance, were identified using a recombinant inbred line (RIL) population derived from the fully resistant line 305E40 (female parent) and the partially resistant line 67/3 (male parent) [44]. Using bulked segregant analysis (BSA) and a de novo genome assembly of 305E40, nine candidate genes in the *FomCH11* region and ten candidate genes in the *FomCH02* region were identified. Notably, the expression levels of seven candidate genes in the *FomCH02* region were induced in eggplant following *Fom* inoculation at 4 and 8 hours, suggesting that these genes may contribute to the full resistance observed in eggplant [44]. Currently, there are no reports regarding pepper or potato resistance to *Foc* and *Fot*. However, with the increasing availability of genome sequences and gapless genome assemblies of pepper, potato, and eggplant cultivars driven by the rapid advancement of sequencing technologies, the identification, cloning, and functional analysis of their respective resistance genes will be significantly expedited.

### Diverse immune receptors utilized in cultivated tomato against *Fol*

I and I-7 are cell surface RLPs. Approximately eighty years ago, extensive screening of wild tomato varieties identified wild tomato *S. pimpinellifolium* accession PI79532 as a source conferring full resistance against *Fol* [121]. The resistance gene from *S. pimpinellifolium* PI79532 was introgressed into cultivated tomato and designated as the *I* gene, which is the first plant resistance gene deployed against Fusarium wilt disease caused by *Fol*. The *I* gene encodes a cell-surface RLP receptor characterized by a leucine-rich repeat (LRR)-capping region, an extracellular LRR region, a loop-out domain rich in serine and threonine residues, and a C-terminal LRR-capping region [122]. The *I* gene is conserved among solanaceous vegetables, including pepper, potato, eggplant, and tomato, but not conserved in *Arabidopsis* and *Brassica* species [122] (Unpublished data). However, the susceptible allele in tomato, designated as *i*, encoding a protein that differs by nine amino acids compared to the I protein, is not able to recognize Avr1 [122]. Like other RLPs, I lacks a cytosolic signaling domain, and therefore requires a signaling partner to initiate downstream immune responses [123]. *I/Avr1* recognition triggers SERK3/BAK1- and SOBIR1-dependent cell death in *N. benthamiana*, indicating that I requires SERK3/BAK1 and SOBIR1 for signal transduction to induce defense responses (Fig. 4) [122]. The specificity of Avr1/I recognition depends on the conserved residues Q922 and T925 of I that are also conserved in the BIR and PSKR1 proteins involved in the interaction with SERK3/BAK1 [122, 124]. Moreover, the central immune regulator enhanced disease susceptibility 1 (EDS1) is genetically required for I-mediated resistance [125]. Consistent with this, EDS1 functions as a key signaling node for resistance mediated by LRR-RLPs and all tested TIR-NB-LRR receptors [122, 126]. Although SERK3/BAK1 and SOBIR1 are required for Avr1/I-dependent cell death in *N. benthamiana*, direct physical interactions between I and these co-receptors have not yet been demonstrated in tomato. Additional studies are needed to confirm the *in planta* association between I and its signaling partners. Historically, a second resistance gene, *I-1*, was derived from *S. pennellii* and was considered nonallelic to *I*, and RFLP analysis positioned *I-1* between the markers TG20 and TG128 on chromosome 7 [127]. However, recent studies found that these *I-1*-linked RFLP markers are absent from chromosome 7 in the tomato line 90E402F that is supposed to carry the *I-1* gene, suggesting that the *I-1* gene may not exist, and these tomato lines in fact carry *I* instead [98]. Another RLP receptor, *I-7*, originating from *S. pennellii* P1414773, confers broad resistance to all known *Fol* races (Fig. 4). Although molecular markers for physical mapping of *I-7* are unavailable, *I-7* was mapped to a small introgression region from *S. pennellii* on chromosome 8 using an RNA-seq approach combined with single-nucleotide polymorphism (SNP) analyses [88]. I-7-mediated resistance against *Fol* is also EDS1-dependent. However, the specific *Fol* avirulence determinant perceived by I-7 to trigger immune responses remains unknown (Fig. 4).

I-2 is a CC-NB-LRR (CNL) intracellular immune receptor. The *I-2* gene was the second tomato wilt resistance gene identified and confers complete resistance against *Fol* race 2. It was introgressed into cultivated tomato from *S. pimpinellifolium* in 1965 [128]. The dominant *I-2* gene was mapped genetically to chromosome 11, and map-based positional cloning subsequently revealed that I-2 encodes a coiled-coil nucleotide-binding leucine-rich repeat (CC-NB-LRR) protein [129]. The NB-ARC domain of I-2 displays ATP-binding activity that is fully dependent on the presence of a divalent cation and ATPase activity that hydrolyzes ATP *in vitro* [130]. Five conserved motifs, including the P-loop, the RNBS-A, the Walker B, the RNBS-D, and the MHD, are present in the NB-ARC domain of I-2 [131, 132]. A mutation in the p-loop results in reduced ATP binding activity, while individual mutations in the RNBS-A and the Walker B motifs impair ATP hydrolysis but not ATP binding [130, 132]. Interestingly, these ATP hydrolysis-deficient mutants cause autoactivation of I-2, leading to a hypersensitive response when transiently expressed in *N. benthamiana*, which requires the intact P-loop motif and its ATP-binding activity [132]. Further studies revealed that heat shock protein 90 (HSP90) and phosphatase 5 physically interact with the LRR domain of I-2. Silencing of *HSP90* in *N. benthamiana* completely abolishes cell death induced by autoactivating I-2 mutants and prevents I-2 protein accumulation, indicating that HSP90 participates in I-2-mediated resistance (Fig. 4) [133, 134]. In addition, required for stability of I-2 (RSI2), a member of the HSP20 family, also interacts with the LRR domain of I-2 [134]. Silencing of RSI2-related HSP20s in *N. benthamiana* impairs the hypersensitive cell death induced by autoactivating I-2 mutants and completely blocks I-2 protein accumulation, suggesting that RSI2 functions as a chaperone required for I-2 stability and activity (Fig. 4) [134]. Avr2 alone is sufficient to trigger I-2-mediated cell death, but the *Avr2*-*SIX5* gene pair is required for *I-2*-mediated disease resistance in tomato plants (Fig. 4) [79, 135].

I-3 is a cell surface-localized RLK. With the rapid evolution and emergence of *Fol* races, efforts to identify resistance genes conferring race-specific immunity from wild and established tomato cultivars are ongoing. Almost thirty years ago, two single dominant genes conferring race-specific resistance to *Fol* race 3 were identified, and both were initially designated as *I-3*. One *I-3* gene was identified from *S. pennellii* accession P1414773 through segregation analysis, while the second *I-3* gene was identified from *S. pennellii* accession LA716 after screening more than 900 wild and cultivated tomato accessions and analyzing their segregation for resistance [136, 137]. Subsequent fine-mapping studies revealed that these two *I-3* genes are different. The gene from LA716 retained the *I-3* designation, whereas the gene from P1414773 was renamed as *I-7* [138]. Detailed fine mapping of the *I-3* locus on chromosome 7 identified three candidate genes encoding the S-receptor-like kinases (SRLKs), each containing an extracellular S-ectodomain, a single transmembrane domain, and a cytoplasmic serine/threonine kinase domain [75]. Further functional assays using transgenic tomato lines confirmed that *SpSRLK5* corresponds to *I-3* and mediates Avr3-dependent resistance against *Fol* race 3 (Fig. 4) [75]. However, co-expression of *I-3* and *Avr3* gives no visible cell death in tobacco leaves [75]. Within the RLK family, both leucine-rich repeat receptor-like kinases (LRR-RLKs) and non-LRR RLKs have been implicated in disease resistance. The SRLKs, including lectin-type RLK I-3 and RFO3, represent a distinct subclass of RLKs involved in plant defense [139].

## Signaling responses in resistant eggplant and tomato activated by *Fo* infection

### *Rfo-sa1* locus-mediated signaling responses upon *Fom* infection

Introgression lines carrying the *Rfo-sa1* locus were challenged with *Fom* at 4 and 8 hours to dissect the signaling responses activated by the *Rfo-sa1* locus [140]. Suppression subtractive hybridization (SSH) and microarray analyses identified that genes encoding PR5 and PR1 proteins, lipoxygenase, and a miraculin-like protein were remarkably upregulated at early time points. Gene Ontology (GO) enrichment analysis further indicated that the differentially expressed genes (DEGs) were enriched in terms associated with the sterol biosynthetic process, hydroxymethylglutaryl-CoA reductase (NADPH) activity, triterpenoid biosynthetic process, and endoplasmic reticulum membrane, suggesting activation of distinct metabolic programs following *Fom* infection. Notably, DEGs assigned to GO categories related to defense responses, oxidation–reduction processes, and lipid metabolic processes displayed significant upregulation at 8 hours following *Fom* inoculation. Consistently, MapMan analysis revealed induction of genes involved in jasmonate signaling, secondary metabolism, redox state maintenance, and the shikimate pathway, while genes associated with auxin and ethylene signaling pathways, transcription factor activity, and heat shock proteins were strongly down-regulated [140]. Taken together, these findings suggest that the activation of *Rfo-sa1* following *Fom* infection amplifies a basal plant immune response in resistant introgression lines.

### Downstream signaling responses activated by tomato R proteins against *Fol*

Upon perception of pathogen- or damage-associated molecular patterns (PAMPs or DAMPs), cell-surface pattern recognition receptors (PRRs) recruit receptor-like cytoplasmic kinases (RLCKs) to activate pattern-triggered immunity (PTI) [141–143]. PTI triggers rapid production of ROS, calcium influx, and mitogen-activated protein kinases (MAPKs)/calcium-dependent protein kinases (CDPKs) cascades, leading to transcriptional reprogramming and the activation of salicylic acid (SA), jasmonic acid (JA), and ethylene (ET) defense pathways [142]. In effector-triggered immunity (ETI), NLR-formed resistosomes induce Ca^2+^ influx or produce NADase-derived signaling molecules, triggering intracellular immune signaling that converges on the EDS1-PAD4 or EDS1-SAG101 complex. This complex coordinates with helper NLRs, such as ADR1 or NRG1, to promote Ca^2+^ channel activation and defense gene transcription [144, 145]. Notably, PTI and ETI share common downstream components, such as calcium influx, MAPK activation, ROS production, and transcriptional reprogramming involving salicylic acid (SA) biosynthesis and defense gene expression [142]. In addition, both can mutually potentiate and require synergistic action to amplify immune responses against diverse pathogens [141]. In tomato, a conceptual overview of these receptor-mediated recognition and signaling pathways is presented in Fig. 4. The cell surface-localized RLP I, requires the co-receptors SOBIR1 and SERK3/BAK1 for signal transduction following recognition of the Avr1 effector, with recognition specificity influenced by polymorphisms in its C-terminal domain [22]. Another LRR-RLP I-7 confers resistance to multiple *Fol* races, and functions in an EDS1-dependent manner [88]. Similarly, the intracellular CC-NB-LRR I-2 protein mediates recognition of Avr2 and also requires the EDS1 signaling hub for full activation of defense responses [129].

In tomato, downstream signaling responses triggered by either surface-localized or intracellular immune receptors against *Fol* remain largely unexplored. Tomato plants carrying the *I* resistance gene activate the jasmonic acid (JA) signaling pathway upon *Fol* infection, accompanied by the biosynthesis of benzenoids and terpenoids and the production of JA-responsive volatiles such as JAZ7, thereby contributing to induced resistance in the host plant [146]. Early activation of JA and ABA signaling pathways has been observed during the incompatible interaction between *Fol* race 1 and *I*-carrying tomato plants, suggesting that antagonistic and synergistic crosstalk between these pathways contributes to the establishment of effective effector-triggered immunity against *Fol* [146]. It has been documented that perception of pathogen elicitors by host cells triggers a rapid burst of ROS, which activates the MAPK signaling cascade, resulting in a broad spectrum of downstream defense responses [147]. Notably, the MAPK cascade serves as a key regulator that connects ROS-mediated signaling with diverse phytohormone pathways, including jasmonic acid (JA), salicylic acid (SA), abscisic acid (ABA), and ethylene (ET), thus enabling dynamic regulation of defense gene expression [148]. Exogenous application of salicylic acid (SA) enhances tomato resistance to *Fol* by alleviating symptoms such as leaf yellowing, wilting, and vascular browning, suggesting the involvement of SA-dependent systemic acquired resistance (SAR) [149]. In contrast, silencing of a tomato gene encoding SA methyltransferase resulted in increased resistance to *Fol*, indicating that suppression of SA methylation enhances tomato resistance to *Fol* [150]. JA signaling also contributes to tomato resistance through interactions with beneficial Trichoderma spp., which enhance JA-mediated defenses [151]. During *Fol* infection, tomato plants accumulate jasmonate ZIM-domain (JAZ) proteins and upregulate specific abscisic acid (ABA) receptor genes, indicating that components of the JA, ET, and ABA pathways collectively regulate defense-related gene expression [152]. Therefore, we can propose that the tomato immune signaling network against *Fol* infection likely integrates multiple shared signaling networks that coordinate ROS production, MAPK activation, and phytohormone signaling. Complex synergistic and antagonistic interactions among JA, SA, ABA, ET, and ROS burst fine-tune defense gene expression, ultimately leading to the induction of pathogenesis-related (PR) proteins, enhanced ethylene biosynthesis, and hypersensitive responses such as localized cell death.

## Synthetic and tomato-driven microbiome-based approaches to inhibit Fusarium wilt disease

The downstream events of host immunity can facilitate the recruitment of beneficial microbiomes, which contribute to the suppression of soil-borne diseases [153]. In turn, soil-borne plant pathogens have evolved strategies, such as effectors, to overcome this microbial barrier and successfully infect the plant [154]. Therefore, beneficial microbial communities in the rhizosphere play a crucial role in plant-pathogen interactions. In fact, the classical disease triangle, consisting of three essential factors including the host, pathogen, and environment, has recently been expanded to incorporate the plant-associated microbiome as an indispensable determinant of disease outcomes and plant health [155]. This paradigm shift recognizes that the microbial communities inhabiting plant surfaces and internal tissues, collectively known as the phyllosphere, rhizosphere, and endosphere, play indispensable roles in shaping plant immunity and disease outcomes. Beneficial microorganisms that reside in these niches can modulate plant defense responses, suppress pathogen proliferation, and enhance the overall disease resistance of the host [156]. Recent advances in microbiome engineering and synthetic biology have opened new frontiers for sustainable management of soil-borne plant diseases. Emerging approaches such as the design of synthetic microbial consortia (SynComs), the use of soil amendments like biochar, and host-driven microbiome recruitment exemplify how the plant microbiome can be intentionally manipulated to enhance resilience against both biotic and abiotic stresses [157]. Currently, microbiome-based strategies for controlling tomato Fusarium wilt have made significant progress, including the deployment of cross-kingdom synthetic microbial communities (SynComs), biochar amendments to reshape root exudates and promote beneficial rhizosphere microbes, and cultivation of resistant tomato cultivars that selectively recruit disease-suppressive microbes upon infection.

**Figure 5:**
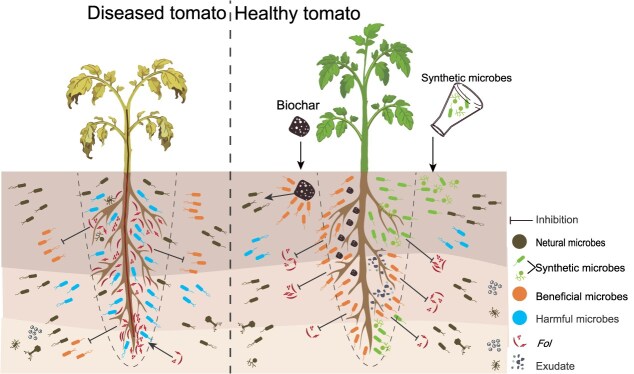
Schematic representation showing the integrated microbiome-based strategy for controlling tomato Fusarium wilt. A diseased tomato plant (left) and a healthy tomato plant (right) under an integrated microbiome-engineering strategy are shown. In the diseased plant, *Fol* infection is associated with a rhizosphere dominated by harmful microbes (blue) and reduced beneficial microbes (orange), leading to severe wilt symptoms. In the healthy plant, biochar (black particles) is applied as an abiotic amendment and cross-kingdom synthetic microbial communities (SynComs; light-green microbes), comprising bacterial and fungal species, are introduced to the rhizosphere. Biochar attracts beneficial microbes (orange) while suppressing harmful populations (blue) and *Fol* (red). SynCom microbes further compete with *Fol* and harmful microbes, collectively promoting a disease-suppressive rhizosphere and maintaining tomato health. In addition, resistant tomato cultivars can secrete exudates to recruit beneficial microbes that can suppress or compete with harmful microbes, thereby promoting tomato resistance against *Fol*.

Based on recent studies, we propose an integrated microbiome-based model for controlling tomato Fusarium wilt. This model unites the host plant, its associated microbial communities, and environmental factors, particularly soil amendments, into a cohesive disease-suppression system. Application of cross-kingdom synthetic microbial communities (SynComs) that combine bacterial and fungal species is a key step in this model [158]. These engineered bacterial and fungal communities overcome single-kingdom assemblages via synergistic interactions that have the ability to compete with pathogens and activate multi-layered plant immunity, thereby enhancing plant immunity and suppressing *Fol* infection (Fig. 5). In parallel, biochar amendments act as abiotic modulators by reshaping the rhizosphere microbiome. By modulating root exudation profiles, biochar indirectly governs microbial recruitment in the rhizosphere, promoting the enrichment of beneficial taxa associated with disease suppression (Fig. 5). This highlights the potential of abiotic-driven microbiome engineering as a complementary strategy to shape plant-microbe interactions and sustain plant health under biotic stress [159]. Moreover, the host genotype plays a pivotal role in shaping these interactions. Resistant tomato cultivars exhibit a pronounced ability to recruit beneficial microbes through secreted exudates in response to pathogen attack, establishing a self-reinforcing defense network. In contrast, susceptible cultivars often display diminished microbial responsiveness, underscoring the importance of host genetic signaling in orchestrating microbiome-mediated immunity [160] (Fig. 5). Understanding these genotype-specific mechanisms may enable targeted breeding strategies that integrate plant genetics with microbiome engineering to achieve durable disease resistance.

## Future perspective

Although meaningful progress in understanding the interaction between solanaceous vegetables and *Fo*, especially in the tomato-*Fol* interaction, has been made, including advances in genome sequencing, effector biology, host immunity, and microbiome-based suppression of Fusarium tomato disease, achieving durable resistance in solanaceous vegetables against *Fo* remains a significant challenge. The lack of high-quality and chromosome-level genome assemblies for *Foc*, *Fot,* and *Fom* hinders the dissection of the molecular mechanisms employed by these pathogens. Recent near-complete *Fol* genome sequences now make it possible to examine, in detail, how mobile chromosomes and fast-changing effector genes contribute to the appearance of new pathogenic races. On the host side, the lack of resistance resources and identified resistance genes in eggplant, pepper, and potato restricts the effective deployment of resistance in breeding. In addition, translating knowledge of tomato immune receptors into durable, broad-spectrum resistance is hindered by extensive *Fol* genetic variation and by the complexity of downstream immune signaling networks. Beyond host- and pathogen-centered strategies, synthetic biology approaches and microbiome-informed managements are emerging as promising components of sustainable disease management frameworks. However, microbiome-based suppression remains constrained by diverse field conditions and limited mechanistic understanding of microbe-microbe and microbe-host interactions. Based on current findings and remaining challenges, future research should focus on addressing the following questions. How does *Fo* population genomics explain virulence diversity, adaptive evolution, and host specificity? How are *Fo* effector genes spatiotemporally regulated during infection? Can RNAi-based targeting of known Fo virulence (effector) genes be used to effectively control Fusarium wilt in both laboratory and field conditions? Can host targets of *Fo* effectors or key susceptibility genes be prime-edited to prevent pathogen manipulation? Can tomato immune receptors be transferred to other solanaceous vegetables with limited resistance resources? Can either the stacking of tomato immune receptors or the use of structure-guided engineered or synthetic immune receptors confer durable resistance to different *Fol* races? Do endophytic microbes contribute to host immunity against *Fo* in solanaceous vegetables? How do tomato immunity and the microbiome interact to shape disease outcomes? Can synthetic microbial communities suppress Fusarium wilt under field conditions? Addressing these questions will be essential for developing durable and broad-spectrum strategies to mitigate Fusarium wilt in solanaceous vegetables.
